# The potential of acetylsalicylic acid and vitamin E in modulating inflammatory cascades in chickens under *lipopolysaccharide*-induced inflammation

**DOI:** 10.1186/s13567-019-0685-4

**Published:** 2019-09-18

**Authors:** Paweł Konieczka, Marcin Barszcz, Paweł Kowalczyk, Michał Szlis, Jan Jankowski

**Affiliations:** 1 Department of Animal Nutrition, The Kielanowski Institute of Animal Physiology and Nutrition, Polish Academy of Sciences, 05-110 Jabłonna, Poland; 2 Department of Poultry Science, University of Warmia and Mazury in Olsztyn, 10-719 Olsztyn, Poland

## Abstract

Distinct enzymes, including cyclooxygenase 1 and 2 (COX-1 and COX-2), lipoxygenase (LOXs), and cytochrome P450 monooxygenase (CYP450), produce different stress mediators and mediate inflammation in birds. Bioactive agents such as acetylsalicylic acid (ASA) and vitamin E (vE) may affect enzyme activities and could be used in poultry production to control the magnitude of acute phase inflammation. Here, we characterized COX, LOX, and CYP450 mRNA expression levels in chicken immune tissues in response to *Escherichia coli* lipopolysaccharide (LPS) challenge and investigated whether ASA and vE could alter gene expression. Additionally, for the first time in chickens, we evaluated oxygen consumption by platelet mitochondria as a biomarker of mitochondria function in response to ASA- and vE. LPS challenge compromised bird growth rates, but neither dietary ASA nor vE significantly ameliorated this effect; however, gradually increasing dietary vE levels were more effective than basal levels. ASA regulated arachidonic acid metabolism, providing an eicosanoid synthesis substrate, whereas gradually increasing vE levels evoked aspirin resistance during challenge. Gene expression in immune tissues was highly variable, indicating a complex regulatory network controlling inflammatory pathways. However, unlike COX-1, COX-2 and CYP450 exhibited increased mRNA expression in some cases, suggesting an initiation of novel anti-inflammatory and pro-resolving signals during challenge. Measuring oxygen consumption rate, we revealed that neither the ASA nor vE levels applied here exerted toxic effects on platelet mitochondria.

## Introduction

Modern-day broilers achieve market weight at an age when their immune system is not fully developed; thus, it is a great challenge for fast-growing birds to cope with pathophysiological disorders during infectious disease. In poultry, physiological and pathological stimuli, including pathogen invasion, trigger activation of the arachidonic acid (AA, 20:4n-6) metabolic cascade, which is controlled by three enzymatic pathways: cyclooxygenase 1 and 2 (COX-1 and COX-2, respectively), lipoxygenase (LOXs), and cytochrome P450 monooxygenase (CYP450) pathways. The activity of the respective enzymes results in the formation of eicosanoids, which are locally acting lipid mediators of inflammation and tissue homeostasis. The COX-derived eicosanoids are prostaglandins (PGs) and thromboxanes (TXs), the LOX-derived eicosanoids are leukotrienes (LTs), and the CYP450-derived eicosanoids are hydroxyeicosatetraenoic acids (HETEs) and epoxyeicosatrienoic acids (EETs). The biosynthesis of eicosanoids is considerably increased in various challenging conditions, and their specific conversion to biologically active products determines the magnitude, duration and nature of subsequent pain perception or inflammatory responses [1].

The recognition of microbial components, such as lipopolysaccharide (LPS), by pattern recognition receptors, constitutes a first-line defence against invading pathogens in birds. Consequently, pathogen stimulation triggers the induction of various signalling pathways, including releasing AA from membrane phospholipids of antigen-presenting cells by the action of phospholipase A_2_ and converting released AA into different groups of eicosanoids [2]. In general, AA-derived eicosanoids act as proinflammatory molecules with pain, fever and acute inflammation manifestations in response to microbial invasion [3]. Given the key role of eicosanoids in the innate response of the host to pathogen challenge, targeting their regulation requires understanding of their complex interactions to develop applicable solutions to avian infection problems [4].

The eicosanoid formation pathway might be affected by dietary lipid manipulations. Elevated levels of dietary n-3 PUFA supported with vitamin E (vE) as an antioxidant exert regulatory actions on the defence system by changing AA metabolism, thereby reducing damage caused to different cell systems by pathogen invasion [5–10]. Another way to target AA-derived eicosanoid biosynthesis during inflammation is the application of non-steroidal anti-inflammatory drugs (NSAIDs), with acetylsalicylic acid (aspirin, ASA) being the most frequently used NSAID in poultry. The regulatory mechanism of ASA is based on the inhibition of the biosynthesis of the most proinflammatory PGs via acetylation of COX-1; thus, ASA can be used to relieve the acute phase during inflammation [1]. More recently, the regulatory actions of ASA have been reported to be more complex and may affect microinflammation through the generation of a novel array of bioactive lipid mediators via sets of enzyme-dependent pathways other than COX-1. Nevertheless, among reports, the advances that continue to emerge primarily relate to human medicine, while data concerning novel mechanisms targeting avian disease problems are scarce.

There is an urgent need in the poultry sector to develop an effective strategy that can limit or prevent infection using dietary intervention. Given that, in this study, we identified the simultaneous actions of COX-, LOX-, and CYP450-enzyme sets mediating the inflammatory response in birds during *E*. *coli* endotoxin challenge. Additionally, we applied for the first time in a chicken model a fluorescence-based analysis of oxygen consumption for the direct determination of platelet mitochondria function. This method was previously applied for use in clinical settings and with a wide range of in vitro models for drug-toxicity assessment [11, 12]. In our opinion, this assay may also have the potential to be used in inflammation-related studies on poultry.

## Materials and methods

### Chicken experiment

The experiment was run on a total of 108 1-day-old female Ross 308 broilers purchased from a local commercial hatchery. The birds were housed in individual cages with free access to pelleted feed and drinking water, and each bird was fed individually and was considered to be a replication. The conditions of the room were maintained according to standard management practices in a commercial chicken house. The birds were fed diets formulated to meet or exceed their nutritional requirement specifications at respective ages [13]. The chickens were divided into six dietary treatments. There was a control group (birds fed a standard commercial diet with a basal vE level—80 mg/kg of diet; placebo). The second treatment (LPS) was the same as the previous one, but the birds were challenged with *E. coli* endotoxin, whereas the third treatment (LPS + ASA) was additionally supplemented with ASA (1 g/kg of diet). Treatments four, five, and six differed from the respective placebo, LPS, and LPS + ASA treatments by applying a gradually increasing level of vE; 160 and 240 mg/kg of diet fed in the periods of 8–21 days and 22–35 days, respectively (Table 1). The birds were reared in the individual cages until day 35 of age. Feed intake was measured, and body weight gain (BWG) was calculated for individual birds.

**Table 1:** **Experimental design of the chicken experiment**

Treatments	Vitamin E level	LPS challenge	Acetylsalicylic acid
Placebo	Basal	–	–
LPS	Basal	+	–
LPS + ASA	Basal	+	+
Placebo	Gradually increasing	–	–
LPS	Gradually increasing	+	–
LPS + ASA	Gradually increasing	+	+

LPS—birds at 32 days of age were intraperitoneally injected at a dose of 250 μg/kg of body weight with *E. coli* LPS; ASA—acetylsalicylic acid at a dose of 1 g/kg of diet; basal vitamin E (vE) level—80 mg/kg of diet; gradually increasing vE level—160 and 240 mg/kg of diet fed in the period 8–21 days and 22–35 days, respectively.

All experimental procedures in this study were approved by the Third Local Animal Experimentation Ethics Committee at the Warsaw University of Life Sciences-SGGW, Poland (resolution no. WAW2/60/2017) and were in accordance with the principles of the European Union and Polish law on animal protection.

### Experimental procedures

#### LPS challenge

At 32 days of age, each bird was weighed after 4 h of feed deprivation (this period of feed withdrawal was used before each weighing in this study). During the subsequent 3 days, at the same time period, birds of the LPS-challenged groups were intraperitoneally injected (the lower abdominal region) at a dose of 250 μg/kg of body weight either with LPS (*Escherichia coli* serotype O55:B5; Sigma Chemical, St. Louis, MO, USA) reconstituted in 0.9% sterile saline (0.5 mg/mL) or the same dose of sterile saline alone (placebo group). At the end of the evaluation period, birds were weighed, and the individual performance indices were calculated for the period of LPS challenge.

#### Quantitative real-time polymerase chain reaction

At 35 days of age, chickens were sacrificed by cervical dislocation, and the abdominal cavity was opened for immune tissue sampling (thymus, spleen and bursa of Fabricius); immune tissues were frozen immediately after collection in liquid nitrogen and then stored in pyrogen-free test tubes at −80 °C until RNA isolation.

Total RNA from the collected tissues was isolated using a Total RNA Mini kit (A&A Biotechnology, Gdynia, Poland) according to the manufacturer’s protocol. The isolated RNA yield was estimated spectrophotometrically (Nanodrop, NanoDrop Technologies, Wilmington, DE), with integrity assessed electrophoretically by separation on a 1.5% agarose gel containing ethidium bromide. For complementary cDNA synthesis, 1500 ng/mL RNA from the selected tissues in a total volume of 20 µL was reverse transcribed using a TranScriba Kit (A&A Biotechnology, Gdynia, Poland) according to the manufacturer’s instructions. Specific primers for chicken (*Gallus gallus*) were designed using Primer 3 software (The Whitehead Institute, Cambridge, MA, USA) and synthesized by Genomed (Warsaw, Poland). The respective primer sequences are shown in Table 2.

**Table 2:** **Genes and primers used in this study**

Gene	Primer	Sequence (5′–3′)	Melting temperature (°C)	Product size (nt)	GenBank access no.
ACTB	Forward	TGTTACCAACACCCACACCC	60.11	110	NM 205518.1
	Reverse	TCCTGAGTCAAGCGCCAAAA	60.18		
COX-1	Forward	GCGCATCAGTAGACCTAGCC	60.32	121	JX160009.1
	Reverse	TGGTATTGTGACAGTGCGGG	60.32		
COX-2	Forward	TGTCCTTTCACTGCTTTCCAT	57.77	84	NM_001167718
	Reverse	TTCCATTGCTGTGTTTGAGGT	58.11		
LOX-12	Forward	CTGATTACGCCGTGCTGGAT	60.53	73	XM_015274997.1
	Reverse	ATTGGGGCACACAGGAATGT	59.89		
CYP450p	Forward	ACCACTTCTGGAAGGAGGGA	59.81	108	D49803.1
	Reverse	CGCTCTCGTAGACACCCAAC	60.46		

ACTB: β-actin, COX-1: cyclooxygenase-1, COX-2: cyclooxygenase-2, LOX-12: lipoxygenase-12, and CYP450p: cytochrome P450.

Real-time qPCR was conducted using a 5× FIREPol EvaGreen qPCR Mix Plus (no ROX; Solis BioDyne, Tartu, Estonia) in a total volume of 15 µL containing 3 µL of Master Mix, 9 µL of RNase-free H_2_O, 2× 0.5 µL of primers (0.5 mM), and 2 µL of cDNA template. Amplification was performed using a Rotor Gene 6000 thermocycler (Corbett Research, Mortlake, Australia) according to the following protocol: one cycle at 95 °C for 15 min (enzyme activation), followed by a PCR including 35 cycles at 95 °C for 5 s (denaturation), 60 °C for 25 s (annealing), and 72 °C for 15 s (elongation), and then one cycle at 72 °C for 7 min (product stabilization). A melting curve was performed over 70–95 °C at 0.5 °C intervals. Negative controls without the cDNA template were included in each reaction. Real-time qPCR for each cDNA sample was performed in duplicate. The identity of PCR products was confirmed by direct sequencing (Genomed, Warsaw, Poland, data not shown).

Relative gene expression was calculated using the comparative quantitation option of Rotor Gene 6000 software 1.7 (Qiagen GmbH, Hilden, Germany) and determined using the Relative Expression Software Tool (2008) according to Pfaffl et al. [14], based on a PCR efficiency correction algorithm developed by Pfaffl et al. [15]. The glyceraldehyde-3-phosphate dehydrogenase (GAPDH), β-actin (ACTB) and peptidylprolyl isomerase C (PPIC) genes were tested as housekeeping genes, and using Best-Keeper software, ACTB was chosen as the best endogenous control gene to normalize gene expression in this study.

#### Enzyme-linked immunosorbent assay

Quantitative determinations of AA and 11-dehydro-thromboxane B_2_ (TBX_2_) in chicken serum were performed spectrophotometrically on a MAXMAT PL multidisciplinary diagnostic platform (Erba Diagnostics France SARL, Montpellier, France) using respective enzyme-linked immunosorbent assay (ELISA) kits (MyBioSource, San Diego, CA, USA) according to the manufacturer’s protocols. Briefly, 12 h post-last challenge at 34 days of life, blood samples were collected from 9 chickens into heparinized test tubes, and serum was separated by centrifugation at 1000 × *g* for 15 min at 2 °C within 30 min of collection. Subsequently, serum samples were frozen at −80 °C until analysis. Each sample was determined in duplicate, and a standard curve was plotted separately for each ELISA plate.

#### Oxygen consumption assay

Whole blood from 9 chickens from each experimental group was collected in an amount of 2 mL into heparinized tubes. Blood was gently mixed to avoid microsphere formation, which could reduce the number of platelets and impair the results, and placed on ice for 2 h to allow sedimentation of individual blood cells. After establishing a visible phase boundary, approximately 150 μL of the upper layer containing the platelets were collected and transferred to new tubes containing 300 μL of 10 mM dimethyl sulfoxide (DMSO) for cryopreservation. Samples were stored at −20 °C for further analysis. Before the analysis, fixed platelets were centrifuged at 3500 ×* g* for 5 min at 2 °C to remove DMSO. Isolated platelets were dissolved in 300 μL of HEPES-buffered Tyrode’s solution (119 mM NaCl, 5 mM KCl, 25 mM HEPES buffer, 2 mM CaCl_2_, 2 mM MgCl_2_, and 6 g/L glucose, adjusted with NaOH to a final pH of 7.4). Measurement of the extracellular oxygen consumption rate by platelet mitochondria was performed on a filter-based multi-mode microplate reader (FLUOstar OPTIMA, BMG Labtech, Offenburg, Germany) using MitoXpress-Xtra HS kits (Luxcel Company, Cork, Ireland) according to the manufacturer’s protocol. Fluorescence intensity measurement was performed in 10-min intervals for 90 min at 37 °C under a sealed environment by overlaying with 100 μL of mineral oil to limit the exchange of oxygen. Tyrode’s buffer with 10 μL of MitoXpress-Xtra HS compound was used as a reference. Each sample was analysed in duplicate. The rate of change in fluorescence signal per minute was calculated for each 10-min interval, and an average signal change per minute during the whole 90-min period was calculated for each chicken.

### Statistical analysis

Data are presented as the mean ± standard error of the mean (SEM) (*n* = 9 for each group). Differences between groups were determined using one-way ANOVA with a least significant difference (LSD) test. The significance level was set at *P* < 0.05. All calculations were performed using STATGRAPHICS Centurion XVI ver. 16.1.03 software.

## Results

### LPS challenge compromised bird growth rate

**Figure 1:**
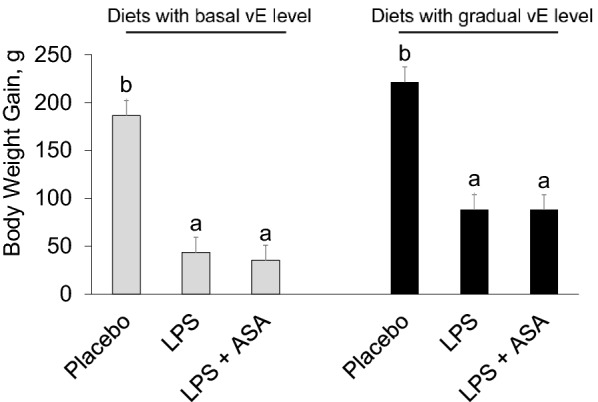
**The body weight gain of chickens in each group.** LPS—birds at 32 days of age were intraperitoneally injected at a dose of 250 μg/kg of body weight with *E. coli* LPS; ASA—acetylsalicylic acid at a dose of 1 g/kg of diet; basal vitamin E (vE) level—80 mg/kg of diet; gradually increasing vE level—160 and 240 mg/kg of diet fed in the period 8–21 days and 22–35 days, respectively. ^a,b^ The different letters represent significant differences (*P* < 0.05). The same letters indicate that the differences between the test group and the placebo group were not significant (*P* > 0.05). Error bars represent the mean values of standard errors for 9 birds in each dietary treatment.

The average BWG for the period of challenge was lower in the LPS groups than in the placebo group (*P* < 0.001). The BWG in challenged chickens fed diets with a basal vE level was almost fivefold lower than that in the respective placebo group, while the BWG in challenged chickens fed a gradually increasing vE level was 2.5-fold lower than that in the respective placebo group. ASA supplementation did not significantly influence bird BWG during the challenge (Figure 1).

### ASA supplementation increased the concentration of AA in the blood

**Figure 2:**
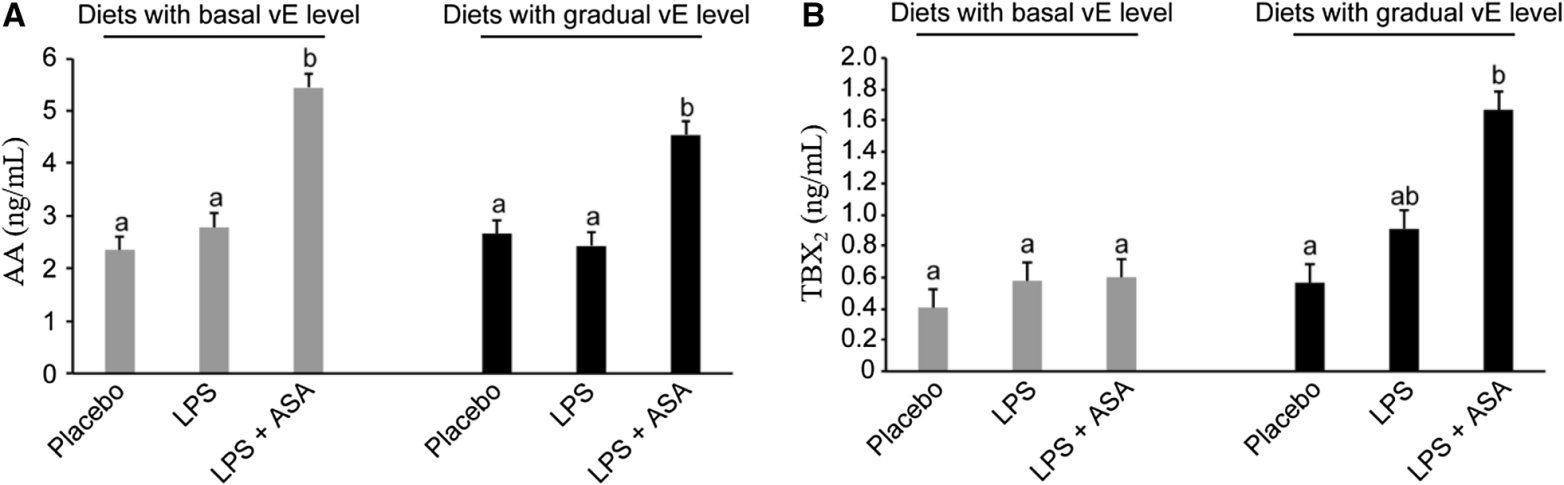
**Blood concentrations of arachidonic acid (AA) (A) and 11-dehydro-thromboxane B2 (TBX**_**2**_**) (B) in chickens of each group.** LPS—birds at 32 days of age were intraperitoneally injected at a dose of 250 μg/kg of body weight with *E. coli* LPS; ASA—acetylsalicylic acid at a dose of 1 g/kg of diet; basal vitamin E (vE) level—80 mg/kg of diet; gradually increasing vE level—160 and 240 mg/kg of diet fed in the period 8–21 days and 22–35 days, respectively. ^a,b^ The different letters represent significant differences (*P* < 0.05). The same letters indicate that the differences between the test group and the placebo group were not significant (*P* > 0.05). Error bars represent the mean values of standard errors for 9 birds in each dietary treatment.

The blood concentration of AA in birds fed basal and gradually increasing vE treatments averaged 3.52 and 3.21 ng/mL, respectively. In both treatments, dietary supplementation with ASA resulted in significantly increased AA concentrations in chicken blood (*P* < 0.001; Figure 2A).

### Gradually increasing level of vE evoked an ASA effect during challenge

The concentration of TBX_2_ in chicken blood is shown in Figure 2B. In chickens fed diets with basal levels of vE, the concentration of TBX_2_ did not differ, but in the other groups that were fed gradually increasing vE treatments, the concentration of TBX_2_ was increased in birds challenged with LPS and receiving ASA supplementation (*P* < 0.05).

### Enzyme mRNA expression

**Figure 3:**
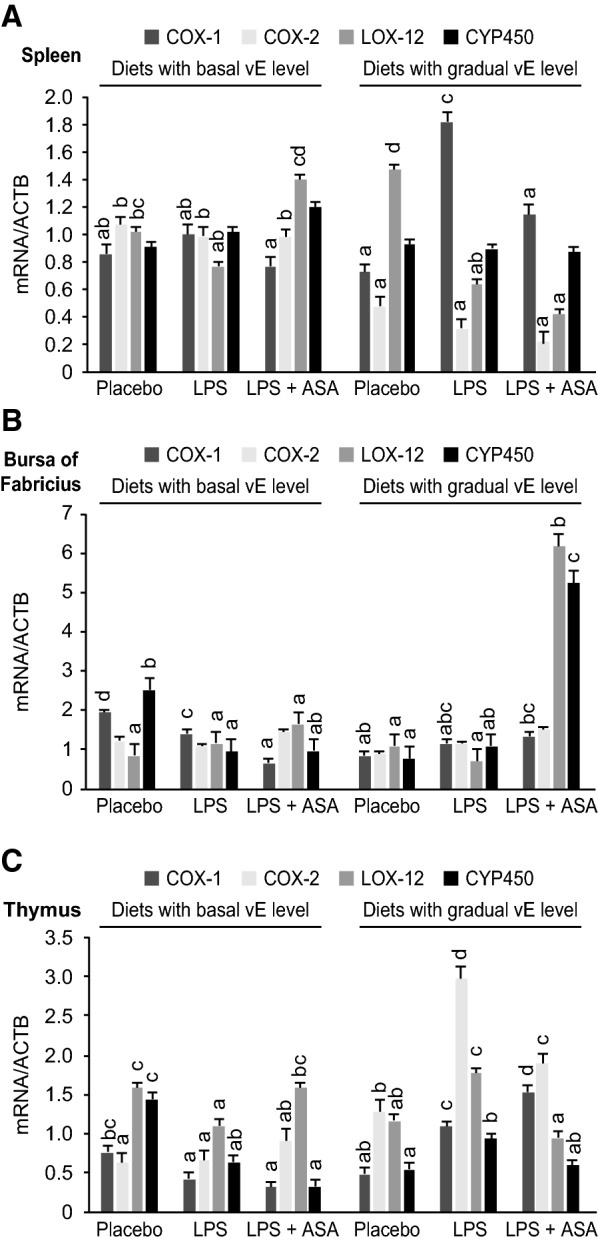
**The expression of COX-1, COX-2, LOX-12, and CYP450 mRNA in the spleen, bursa of Fabricius, and thymus tissues of chickens.** LPS—birds at 32 days of age, for the subsequent 3 days, were intraperitoneally injected at a dose of 250 μg/kg of body weight with *E. coli* LPS, acetylsalicylic acid (ASA) at a dose of 1 g/kg of diet, and a basal level of vitamin E (vE) at a dose of 80 mg/kg of diet; the vE dose was gradually increased to 160/240 mg/kg of diet during the periods of 8–21 days and 22–35 days, respectively. ^a,b,c,d^ The different letters represent significant differences (*P* < 0.05). The same letters indicate that the differences between the test group and the placebo group were not significant (*P* > 0.05). Error bars represent the mean values of standard errors for 9 birds in each dietary treatment.

The mRNA expression of the COX-1, COX-2, LOX-12 and CYP450 in immune tissues is presented in Figure 3. The mRNA expression of the respective enzymes varied in different immune tissues. In the spleen, an increased level of COX-1 was found in the challenged group fed a gradually increasing level of vE, while an increased expression of COX-2 occurred in all groups fed a basal vE level (*P* < 0.001). In the bursa of Fabricius, the highest expression of COX-1 occurred in the placebo group with a basal vE level, and COX-2 expression did not differ significantly, whereas the highest expression of CYP450 occurred in the LPS-challenged group fed a gradually increasing vE level with ASA (*P* < 0.001). Thymus expression of COX-1 and COX-2 was the highest in challenged birds fed a gradually increasing vE level, while CYP450 mRNA expression was the highest in the placebo group with a basal vE level (*P* < 0.001). Feeding birds with a gradually increasing level of vE and with ASA resulted in LOX-12 mRNA expression in the spleen and thymus being lower than that in the placebo group (*P* < 0.001), while in the bursa of Fabricius, the mRNA expression of LOX-12 was at the same level in all groups apart from the group that was fed a gradually increasing vE level and treated with LPS + ASA (*P* < 0.001).

### ASA counteracted platelet mitochondrial dysfunction during LPS challenge

**Figure 4:**
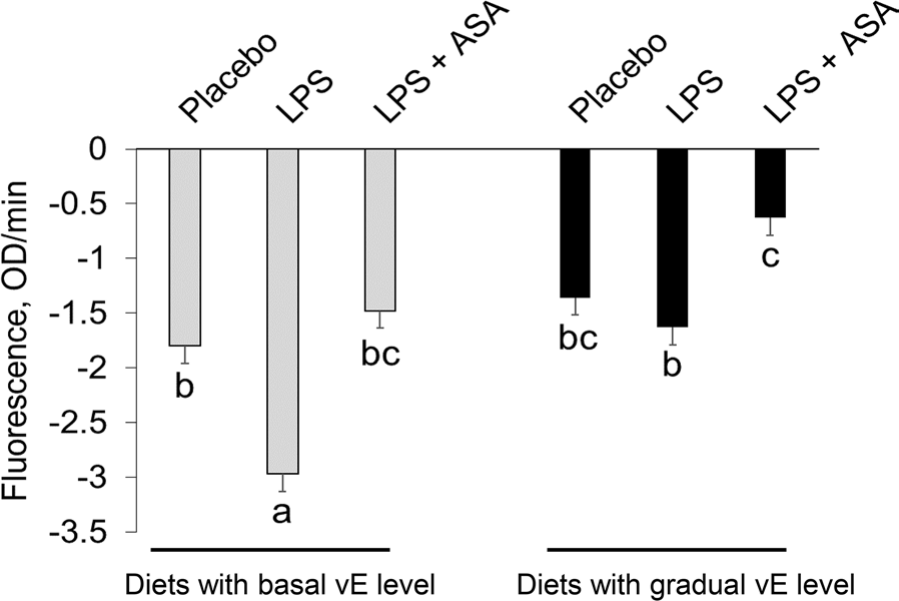
**Oxygen consumption rate by platelet mitochondria expressed as a rate of fluorescence signal change per minute.** LPS—birds at 32 days of age, for the subsequent 3 days, were intraperitoneally injected at a dose of 250 μg/kg of body weight with *E. coli* LPS, acetylsalicylic acid (ASA) at a dose of 1 g/kg of diet and a basal level of vitamin E (vE) at 80 mg/kg diet; the vE dose was gradually increased to 160 and 240 mg/kg of diet during the periods of 8–21 days and 22–35 days, respectively. ^a,b,c^ The different letters represent significant differences (*P* < 0.05). The same letters indicate that the differences between the test group and the placebo group were not significant (*P* > 0.05). Error bars represent the mean values of standard errors for 9 birds in each dietary treatment.

The oxygen consumption rate by the platelet mitochondria, expressed as a change in fluorescence signal in time (intensity inversely proportional to the extracellular oxygen level) is presented in Figure 4. In all groups, the fluorescence signal decreased in time. In birds fed a basal vE level, the rate of signal decrease was greater in the LPS group than in the placebo and LPS + ASA groups; in birds fed diets with gradually increasing vE levels, the rate of signal decrease was lower in the LPS + ASA group than in the LPS group (*P* < 0.001).

## Discussion

Recently, a number of strategies have been investigated towards anti-inflammatory effect. Our previous work [16] showed that although feeding broilers diets high in n-3 fatty acids (FAs) and with an increased vE dose clearly limited the availability of AA and consequently antagonised lipid peroxidation during mitogen stimulation. This study provides evidence that the specific response in challenge conditions in birds is mediated by the simultaneous action of different enzymes, which produce lipid hormones involved in inflammation. In a recent study, we investigated the concept that ASA might selectively inhibit COX-1 without blocking other enzymes, which could result in the downregulation of highly proinflammatory PG formation while promoting the biosynthesis of mediators of anti-inflammatory properties. Owing to the prooxidant activity of excessive dietary vE [17], we additionally investigated the potential of a gradually increasing vE dose to support ASA actions.

The *E. coli* endotoxin challenge applied in this study was successful at inducing overall inflammation in birds. This effect was manifested in the lower BWG in the LPS-challenged groups than that in the placebo group. Interestingly, we observed a growth depression after the challenge period in some cases, indicating a high magnitude of inflammation. A similar response was observed in other reports [2, 18]; hence, it was found that the administration of LPS to birds triggered the repartitioning of nutrients away from growth maintenance to mediate inflammatory processes via the neuroendocrine-immune axis. According to Klasing [19], the costs of the immune response in birds consist of 9% of the nutrient needs; however, constant maintenance of activated cell populations and production of constitutively expressed inflammatory mediators might require much greater costs than those required for maintenance [20]. Our results demonstrated the costs associated with mounting an immune response as a result of challenge.

**Figure 5:**
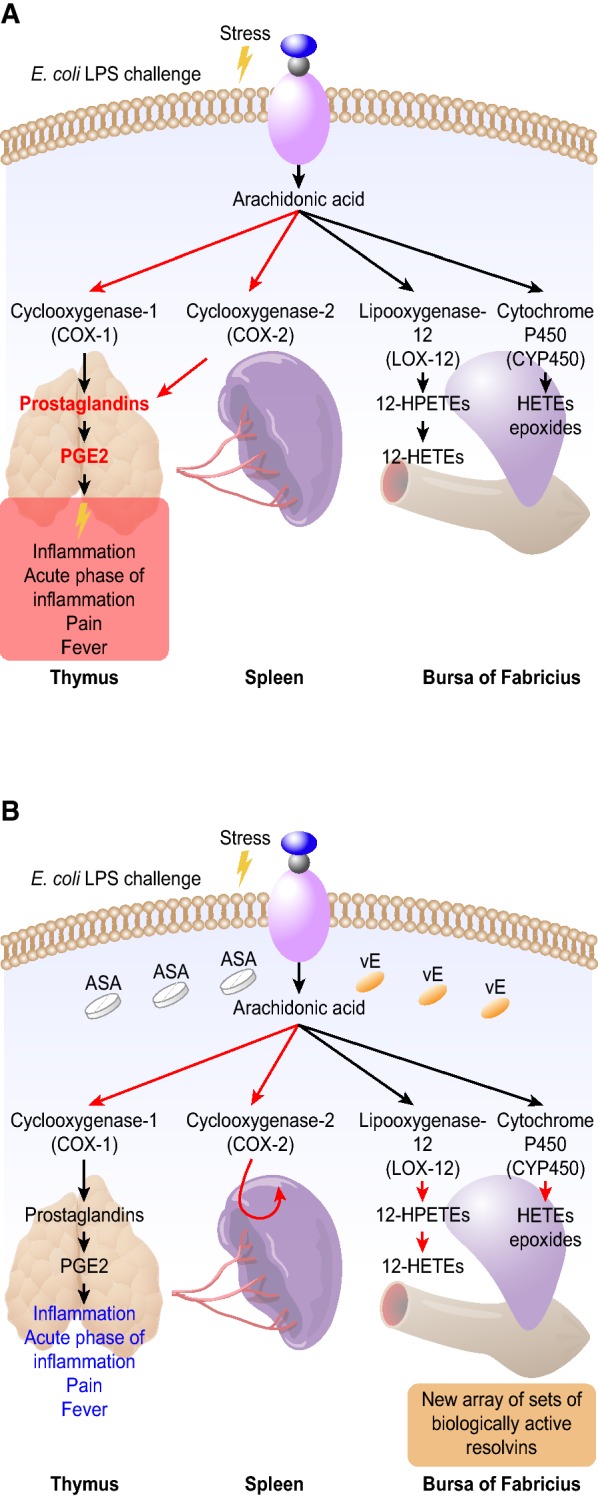
**Possible pathways for the synthesis of bioactive stress mediators from the arachidonic acid (AA) cascade in the immune tissues of chickens.** In response to *E. coli* LPS stimuli, AA is released from membrane phospholipids. It is then converted to biologically active series of prostaglandins (PGs) including prostaglandin E2 (PGE2), by the action of cyclooxygenase-1 (COX-1) and cyclooxygenase-2 (COX-2) enzymes, which evoke the acute phase of inflammation (A). The stress mediator synthesis pathway differs in the presence of acetylsalicylic acid (ASA) and vitamin E (vE) (B); COX-1 is inhibited while, COX-2 is irreversibly acetylated and switches from producing PGs. In such circumstances, AA is metabolized to a greater extent by lipoxygenase-12 (LOX-12) and cytochrome P450 (CYP450) pathways. This mechanism, in turn, may lead to the generation of novel biologically active resolvins via pathways other than those mediated by COX-1. The generation of these and related compounds represents a novel mechanism for the therapeutic benefits of dietary supplementation with ASA and vE, which may be important in controlling inflammation in birds.

Feeding broilers with a typical commercial diet provides AA as a dominant substrate for eicosanoid synthesis [9]. An inflammation process triggers the release of AA from membrane phospholipids, and via phospholipase A_2_, AA becomes available as a substrate for eicosanoid formation. Therefore, the total available pool of blood AA may have considerable consequences for the inflammatory response pathway. The results of our study indicate the regulatory action of ASA in mediating AA metabolism. In both challenged groups but not in those fed ASA, the blood level of AA was the same as that in the placebo group. This result may suggest that during LPS challenge, AA conversion into stress mediators was maintained at a constant rate while ASA supplementation inhibited enzymes responsible for AA conversion. Thus, an increased level of AA in the ASA-fed groups was observed. These findings may indicate that ASA action in birds could inhibit stress mediator synthesis. This phenomenon might be of particular importance since it was shown in mice [21] that inhibition of the enzymatic pathway by ASA generated a novel array of bioactive lipid mediators, which provides a novel target for therapeutic action Figure 5.

Currently, the measurement of blood TBX_2_ concentration is considered the best laboratory approach to study aspirin resistance. COX-1 metabolizes AA into thromboxane A_2_, which in turn, due to a short half-life, is converted into TBX_2_ (stable metabolite). ASA functions by acetylating and irreversibly inhibiting COX-1, therefore inhibiting the production of TBX_2_, which might be a direct way to analyse aspirin’s resistance. In this context, our results showing increased levels of blood TBX_2_ in challenged birds fed a gradually increasing vE level are unexpected. It is known that vE prevents AA oxidation [24]; therefore, a higher pool of AA is able to enter the eicosanoid pathway during inflammation, which may explain the obtained results. The possible mechanism of this phenomenon is not clear, but it might be true that the biosynthesis of TBX_2_ in these birds occurred by pathways that are not blocked by aspirin, e.g., by COX‐2 [22]. This hypothesis corresponds to our speculation raised above that ASA may generate a novel, aspirin-triggered array of bioactive stress mediators through a shift in enzymatic complex activity. However, an increased level of blood TBX_2_, but in only challenged birds, seems to exclude the gradually increasing level of vE contributing via oxidative stress to aspirin resistance [23] since we did not observe an increased TBX_2_ level in the respective placebo group. The fact that vE is known to prevent AA oxidation might be significant [24]; therefore, a higher pool of AA is able to enter the eicosanoid pathway during inflammation, which was also evidenced in the recent study.

Although inflammatory lipid mediators are produced mainly by activated macrophages and monocytes as a response to stressor stimuli, the contribution of the other pathways of their supply in avian species is poorly discovered. Indeed, the enzymatic systems of controlling the pathways of inflammatory lipid mediator synthesis are active throughout the tissues of the whole biological system (reviewed by Panigrahy et al. [25]). Understanding this contribution might be of significance and may provide new opportunities to control the inflammation process in birds. We showed that variable mRNA expression of COX-1, COX-2, LOX-12, and CYP450 in immune tissues, including the spleen, bursa of Fabricius and thymus, occurred in birds as a result of dietary treatments and LPS challenge. To the best of our knowledge, there are no data available regarding such gene expression in the bird immune tissues; therefore, we cannot confirm these results with other reports. However, our findings indicated that induced inflammation affected enzyme mRNA expression in bird immune tissues, which was likely triggered via the neuroendocrine-immune axis [26]. Of significance would be understanding whether such a phenomenon occurs similar to that in activated macrophages and monocytes or occurs via an independent pathway and therefore might be a target for modulation. Because the mRNA expression of enzymes was different among immune tissues, we could not observe any logical regulatory mechanism governing this process. Interestingly, we found that the highest mRNA expression of CYP450 in the bursa of Fabricius occurred in challenged birds fed a gradually increasing level of vE and ASA, suggesting a shift in the enzymatic activity towards synthesis of eicosanoids other than PGs, which is mediated by COX-1. Chiang et al. [27] reported that due to the activity of CYP450, a series of aspirin-triggered lipoxins are generated during the inflammatory response. These lipoxins might evoke potent anti-inflammatory and pro-resolving actions. More recently, it was shown in a different model that ASA acetylated COX-2, which led to the inhibition of prostaglandin E_2_, one of the most proinflammatory eicosanoids [28]; furthermore, a novel array of stress mediators was synthesized as a response to inflammation [29]. Szczeklik et al. [30] reported that activated COX-2 releases 15-hydroperoxide of eicosatetraenoic acid, which is a potential substrate for less-proinflammatory eicosanoid mediators, e.g., 15-epi-lipoxins. In line with this finding, our study demonstrated increased COX-2 mRNA expression in the thymus of birds fed a gradually increasing vE level, suggesting that vE may play a regulatory role in mediating the LPS-induced inflammatory response, which corresponds to the findings of another study [31]. In the present study, we observed reduced expression of LOX-12 mRNA in the spleen and thymus. This enzyme plays a key role in various inflammatory pathways and has the ability to oxidise biomembranes, including mitochondrial membranes [32]. According to Mabalirajan et al. [33], LOX can metabolize AA to produce different stress mediators, e.g. PGs, and vE can also play a role in this phenomenon since it reduced the expression of 12/15-LOX and its metabolites in a murine model. Moreover, in the aforementioned study, vE administration restored mitochondrial function in induced inflammation.

In the present study, an oxygen consumption assay was applied to evaluate whether diets with ASA could have toxicity impacts in chickens and ASA interacted with a gradually increasing level of vE. Dietary vE is predominantly mobilized in mitochondrial fractions and is a key element responsible for maintaining their integrity and functions [34, 35]. Therefore, any imbalance of vE in mitochondrial fractions evokes mitochondrion-derived oxidative stress, which consequently impairs mitochondrial function [36]. Oxygen consumption assay allows the determination of respiration rates for metabolic characterization and the evaluation of toxic effects of treatments on mitochondrial function [12]. A lower extracellular level of oxygen indicates a higher rate of its consumption by the mitochondria. Because mitochondria play a key role in metabolic processes of the whole biological system, their impairment is a major mechanism of drug-induced toxicity. In a recent study, we measured the oxygen consumption rate by blood platelet mitochondria as an indicator of the effect of a treatment, as it may have an implication for further application. Blood-based analyses are an easy-to-apply approach and therefore can be used to evaluate dietary treatment effects in birds. Our findings indicated that LPS-induced inflammation increased the rate of fluorescence signal decrease, particularly in birds fed diets with a basal vE level, but this effect was alleviated by ASA. It must be noted that the fluorescence signal from MitoXpress-Xtra HS should increase with time. For unknown reasons, the signal decreased in all birds with time, which may suggest an oxygen leak from blood platelets. Nonetheless, our results indicate that dietary ASA supplementation may have a beneficial effect on platelet mitochondria, while dietary vE level has no impact on extracellular oxygen consumption. These results correspond to previous result showing that LPS challenge compromised bird performance, which suggests that measurement of blood platelet function based on the oxygen consumption assay reflects the health status of chickens. Thus, our findings indicated that oral ASA supplementation did not induce toxicity in birds since the level of extracellular oxygen was higher in the LPS + ASA group than that in the LPS-challenged group. More specifically, we found that blood platelet function in challenged birds that were fed a gradually increasing vE level were restored after ASA supplementation, while a gradually increasing vE level effectively maintained platelet function since we observed a reduced oxygen metabolism efficacy in only LPS-treated birds fed a basal vE level. Taken together, these data suggest that neither ASA nor vE levels applied in this study exerted toxicity effects. Moreover, alleviated mitochondrial dysfunction due to ASA and vE administration might be beneficial during the initial inflammatory response since it was suggested that they play a key function during antigen presentation [37, 38].

In summary, the results of the present study indicate that a complex regulatory system is triggered by *E. coli*-induced inflammation to facilitate the stress-mediator response in birds. Here, we demonstrated that during inflammation, the immune tissue responses at the transcriptional level shift in the gene expression of enzymatic complexes, generating less-proinflammatory eicosanoids. However, the contribution of these pathways to the whole immune response to challenge remains unknown. Our results suggest that ASA plays a regulatory role in stress mediation by increasing the total pool of AA entering eicosanoid pathways and by inhibiting COX-1 expression. The results also suggest that neither ASA nor vE manifested a toxicity effect on platelet mitochondria but feeding birds a gradually increasing level of vE might be associated with ASA resistance during challenge. These findings will likely be beneficial in developing targeted control strategies and/or for monitoring treatment effects.

## Data Availability

The datasets used and/or analysed during the current study are available from the corresponding author upon reasonable request.

## Abbreviations

AA: arachidonic acid
ASA: acetylsalicylic acid
BWG: body weight gain
COX-1: cyclooxygenase 1
COX-2: cyclooxygenase 2
CYP450: cytochrome P450
EETs: epoxyeicosatrienoic acids
FAs: fatty acids
HETEs: hydroxyeicosatetraenoic acids
LOX: lipoxygenase
LPS: lipopolysaccharide
LTs: leukotrienes
PGs: prostaglandins
TBX_2_: 11-dehydro-thromboxane B2
TXs: thromboxanes
vE: vitamin E

## References

[1] Harizi H, Corcuff JB, Gualde N (2008). Arachidonic-acid-derived eicosanoids: roles in biology and immunopathology. Trends Mol Med.

[2] de Boever S, Croubels S, Meyer E, Sys S, Beyaert R, Ducatelle R, de Backer P (2009). Characterization of an intravenous lipopolysaccharide inflammation model in broiler chickens. Avian Pathol.

[3] Calder PC (2006). Polyunsaturated fatty acids and inflammation. Prostaglandins Leukot Essent Fatty Acids.

[4] Kaiser P (2010). Advances in avian immunology–prospects for disease control: a review. Avian Pathol.

[5] Qureshi MA, Gore AB (1997). Vitamin E exposure modulates prostaglandin and thromboxane production by avian cells of the mononuclear phagocytic system. Immunopharmacol Immunotoxicol.

[6] Klasing KC (1998). Nutritional modulation of resistance to infectious diseases. Poult Sci.

[7] Sijben JWC, Klasing KC, Schrama JW, Parmentier HK, van der Poel JJ, Savelkoul HFJ, Kaiser P (2003). Early in vivo cytokine genes expression in chickens after challenge with *Salmonella typhimurium* lipopolysaccharide and modulation by dietary *n*−3 polyunsaturated fatty acids. Dev Comp Immunol.

[8] Hall JA, Jha S, Skinner MM, Cherian G (2007). Maternal dietary n-3 fatty acids alter immune cell fatty acid composition and leukotriene production in growing chicks. Prostaglandins Leukot Essent Fatty Acids.

[9] Cherian G (2007). Metabolic and cardiovascular diseases in poultry: role of dietary lipids. Poult Sci.

[10] Swiatkiewicz S, Arczewska-Wlosek A, Jozefiak D (2015). The relationship between dietary fat sources and immune response in poultry and pigs: an updated review. Livest Sci.

[11] Porceddu M, Buron N, Roussel C, Labbe G, Fromenty B, Borgne-Sanchez A (2012). Prediction of liver injury induced by chemicals in human with a multiparametric assay on isolated mouse liver mitochondria. Toxicol Sci.

[12] Hynes J, Nadanaciva S, Swiss R, Carey C, Kirwan S, Will Y (2013). A high-throughput dual parameter assay for assessing drug-induced mitochondrial dysfunction provides additional predictivity over two established mitochondrial toxicity assays. Toxicol In Vitro.

[13] Aviagen (2014) Ross 308 Broiler: performance objectives. http://en.aviagen.com/assets/Tech_Center/Ross_Broiler/Ross308BroilerNutritionSpecs2014-EN.pdf. Accessed 13 April 2019

[14] Pfaffl MW, Horgan GW, Dempfle L (2002). Relative expression software tool (REST) for group-wise comparison and statistical analysis of relative expression results in real-time PCR. Nucleic Acids Res.

[15] Pfaffl MW, Tichopad A, Prgomet C, Neuvians TP (2004). Determination of stable housekeeping genes, differentially regulated target genes and sample integrity: BestKeeper–excel-based tool using pair-wise correlations. Biotechnol Lett.

[16] Konieczka P, Barszcz M, Chmielewska N, Cieślak M, Szlis M, Smulikowska S (2016). Interactive effects of dietary lipids and vitamin E level on performance, blood eicosanoids, and response to mitogen stimulation in broiler chickens of different ages. Poult Sci.

[17] Konieczka P, Barszcz M, Choct M, Smulikowska S (2018). The interactive effect of dietary n−6: n−3 fatty acid ratio and vitamin E level on tissue lipid peroxidation, DNA damage in intestinal epithelial cells, and gut morphology in chickens of different ages. Poult Sci.

[18] Zheng XC, Wu QJ, Song ZH, Zhang H, Zhang JF, Zhang LL, Zhang TY, Wang C, Wang T (2016). Effects of Oridonin on growth performance and oxidative stress in broilers challenged with lipopolysaccharide. Poult Sci.

[19] Klasing KC (2007). Nutrition and the immune system. Br Poult Sci.

[20] Kogut MH (2009). Impact of nutrition on the innate immune response to infection in poultry. J Appl Poult Res.

[21] Serhan CN, Clish CB, Brannon J, Colgan SP, Chiang N, Gronert K (2000). Novel functional sets of lipid-derived mediators with antiinflammatory actions generated from omega-3 fatty acids via cyclooxygenase 2-nonsteroidal antiinflammatory drugs and transcellular processing. J Exp Med.

[22] Rocca B, Secchiero P, Ciabattoni G, Ranelletti FO, Catani L, Guidotti L, Melloni E, Maggiano N, Zauli G, Patrono C (2002). Cyclooxygenase-2 expression is induced during human megakaryopoiesis and characterizes newly formed platelets. Proc Natl Acad Sci U S A.

[23] Cipollone F, Ciabattoni G, Patrignani P, Pasquale M, Di Gregorio D, Bucciarelli T, Davi G, Cuccurullo F, Patrono C (2000). Oxidant stress and aspirin-insensitive thromboxane biosynthesis in severe unstable angina. Circulation.

[24] Khan RU, Rahman ZU, Nikousefat Z, Javdani M, Tufarelli V, Dario C, Selvaggi M, Laudadio V (2012). Immunomodulating effects of vitamin E in broilers. Worlds Poult Sci J.

[25] Panigrahy D, Kaipainen A, Greene ER, Huang S (2010). Cytochrome P450-derived eicosanoids: the neglected pathway in cancer. Cancer Metastasis Rev.

[26] Mashaly MM, Trout JM, Hendricks G, Al-Dokhi LM, Gehad A (1998). The role of neuroendocrine immune interactions in the initiation of humoral immunity in chickens. Domest Anim Endocrinol.

[27] Chiang N, Arita M, Serhan CN (2005). Anti-inflammatory circuitry: lipoxin, aspirin-triggered lipoxins and their receptor ALX. Prostaglandins Leukot Essent Fatty Acids.

[28] Zendehdel M, Baghbanzadeh A, Yeganeh B, Hassanpour S (2015). The role of cyclooxygenase inhibitors in lipopolysaccharide-induced hypophagia in chicken. Czech J Anim Sci.

[29] Serhan CN, Hong S, Gronert K, Colgan SP, Devchand PR, Mirick G, Moussignac RL (2002). Resolvins: a family of bioactive products of omega-3 fatty acid transformation circuits initiated by aspirin treatment that counter proinflammation signals. J Exp Med.

[30] Szczeklik A, Musiał J, Undas A, Sanak M (2005). Aspirin resistance. J Thromb Haemost.

[31] Zhang X, Zhong X, Zhou Y, Wang G, Du H, Wang T (2010). Dietary RRR-alpha-tocopherol succinate attenuates lipopolysaccharide-induced inflammatory cytokines secretion in broiler chicks. Br J Nutr.

[32] Chanez P, Bonnans C, Chavis C, Vachier I (2002). 15-lipoxygenase: a Janus enzyme?. Am J Respir Cell Mol Biol.

[33] Mabalirajan U, Aich J, Leishangthem GD, Sharma SK, Dinda AK, Ghosh B (2009). Effects of vitamin E on mitochondrial dysfunction and asthma features in an experimental allergic murine model. J Appl Physiol.

[34] Bjorneboe A, Bjorneboe GE, Drevon CA (1990). Absorption, transport and distribution of vitamin E. J Nutr.

[35] Li X, May JM (2003). Location and recycling of mitochondrial alpha-tocopherol. Mitochondrion.

[36] Zhang JG, Nicholls-Grzemski FA, Tirmenstein MA, Fariss MW (2001). Vitamin E succinate protects hepatocytes against the toxic effect of reactive oxygen species generated at mitochondrial complexes I and III by alkylating agents. Chem Biol Interact.

[37] Raby BA, Klanderman B, Murphy A, Mazza S, Camargo CA, Silverman EK, Weiss ST (2007). A common mitochondrial haplogroup is associated with elevated total serum IgE levels. J Allergy Clin Immunol.

[38] del Prete A, Zaccagnino P, Di Paola M, Saltarella M, Celis C, Nico B, Santoro G, Lorusso M (2008). Role of mitochondria and reactive oxygen species in dendritic cell differentiation and functions. Free Radic Biol Med.

